# Study on the antioxidant activity of peptides from soybean meal by fermentation based on the chemical method and AAPH‐induced oxidative stress

**DOI:** 10.1002/fsn3.3612

**Published:** 2023-08-28

**Authors:** JuanJuan Ma, Keying Su, Meimei Chen, Shuo Wang

**Affiliations:** ^1^ Guangzhou College of Technology and Business Guangzhou China

**Keywords:** antioxidant activity, erythrocyte hemolysis, fermentation, peptides, soybean meal, ultrafiltration

## Abstract

Preparation and antioxidant activities of soybean peptides using solid fermentation to decrease the content of trypsin inhibitor (TI) and antigen protein were investigated in this study. The results showed the optimal fermentation conditions were as follows: fermentation time 48 h, the ratio of material to solvent 1:2, inoculum size 12%, and the ratio of Lactic acid bacteria and Aspergillus oryzae 2:1. The hydrolysate was were divided into four components of <1, 1–3, 3–5, and >5 kDa by ultrafiltration based on molecular weight, and the <1 kDa peptides expressed the highest antioxidant activities. Meanwhile, the cell antioxidant activity of the <1 kDa soybean peptides was investigated using AAPH‐induced erythrocyte hemolysis, which effectively inhibited erythrocyte hemolysis with the inhibit rate of 85.8% through inhibition of the ROS intracellular generation. In addition, soybean peptides could significantly restore the intracellular antioxidant enzymes (SOD, GSH‐Px, and CAT) activities, as well as inhibited intracellular MDA generation and depletion of GSH. The intracellular antioxidant detoxifying mechanism of soybean peptides was associated with both non‐enzymatic and enzymatic defense systems. According to this study, fermentation could effectively improve the antioxidant activities of soybean peptides.

## Graphical abstract

Soybean peptides were prepared by fermentation with mixed Lactic acid bacteria and Aspergillus oryzae from soybean meal, meanwhile degrading the macromolecule protein and some antinutritional factors in soybean meal. The soybean peptides expressed the strong antioxidant activity in vitro and intracellular., which can developed as the additive of functional foods, medicines and cosmetic.

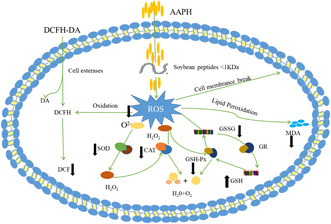

## 1 INTRODUCTION

Soybean had a long history of serving as a food crop and economic commodity, which was widely planted in the world for the nutritive constituents such as proteins (40–50%), carbohydrates (26–30%), lipids (20–30%), vitamins, and mineral substance (Shang et al., 2016). Soybean meal has the same protein as the byproduct of soybean, which was the most important source of protein for animals due to its excellent functional property and physiological activity (Wang, Chen, et al., 2017; Wang, Lei, et al., 2017; Wang, Wang, et al., 2017; Wen et al., 2022). According to the present studies, the protein has the functions of antioxidant, antihypertensive, antitumor, antidiabetic, antibacterial and prevent atherosclerosis, which was associated with oxidative stress (Agyei, 2015; Carmen et al., 2019; Sanjukta & Rai, 2016; Yang et al., 2011; Yuan et al., 2017). However, the soybean protein was difficult in digestion and absorption for its complicated structure and macromolecule sometimes. Soybean peptides have drawn the attention of researchers based on the properties of antioxidant in scavenging the free radical (Liu et al., 2014; Sila & Bougatef, 2016). Antioxidant peptides can protect human body from damages induced by reactive oxygen species (ROS) generated under the stimulation of external factors (Asoodeh et al., 2016; Kong et al., 2023; Sheng et al., 2023). According to the present reports, the activities of soybean peptides mainly depend on the peptide chain length, amino acids composition and amino acids sequence. Generally, the soybean peptides consist of 2–20 amino acids with low molecular weight to be considered to be healthy and nutritious (Sarmadi & Ismail, 2010). Amino acids, such as Trp, Tyr, Gly, His, Ala, Phe, Met, Leu, and Val have been reported to be the components of antioxidant peptides (Cai et al., 2022; Liu et al., 2016; Sun et al., 2022). Some reports have described the production of soybean peptides according to the hydrolysis of protein through the enzyme hydrolysis and fermentation, which are attribute to the function of enzymes whether using enzyme directly or fermentation (Abeyrathne et al., 2016; Ranamukhaarachchi et al., 2013; Ren & Li, 2022). Fermentation of soybean meal mainly depends on the proteolytic system by the microorganisms involved during fermentation by the strains like lactic acid bacteria, aspergillus oryzae, and bacillus subtilis (Dai et al., 2017, 2021; Naifu et al., 2014; Suprayogi et al., 2022). It has been shown that fermentation soybean meal increased the antioxidant activity with the mixture proteolytic microorganisams. During fermentation, complex compounds are broken down into small molecules by microorganisms, which can improve nutritional and functional properties of raw materials (Bi et al., 2015). Besides, some antinutritional components in soybean meal are degraded such as trypsin inhibitor (TI), phytic acid, urease, antigen‐protein, oligosaccharide (hydrothreose and raffinose), which improve the nutritional and functional properties of soybean meal (Coscueta et al., 2017; Feng, Liu, Xu, Liu, & Lu, 2007; Feng, Liu, Xu, Lu, & Liu, 2007; Hassaan et al., 2015).

In this study, soybean peptides were prepared by solid fermentation and the optimal fermentation conditions were optimized by response surface methodology. After fermentation, primary purification was performed by ultrafiltration with different ultrafiltration membranes of 1, 3, and 5 kDa molecular weight to obtain different peptide fractions. The antioxidant activity of soybean peptides was evaluated based on the method of chemistry and protecting from AAPH‐induced erythrocyte oxidative hemolysis, which regarded scavenging rate of free radicals, ROS content in cells, and enzymes activities such as glutathione peroxidase (GSH‐Px), catalase (CAT), and superoxide dismutase (SOD) as the index. The study can provide theoretical basis for development and utilization of soybean meal in functional foods in further study.

## 2 MATERIALS AND METHODS

### 2.1 Materials

Soybean meal were purchased from market. *Lactic acid bacteria and Aspergillus oryzae* (L6) were collected from laboratory. MRS and PDA medium were purchased from Huankai microbiological technology company (Guangzhou). The soybean glycinin and β‐conglycinin Elisa kits were purchased from Beijing. DPPH, ABTS, AAPH, and vitamin C were from Sigma company (USA). The kits for determination GSH‐Px, SOD, MDA, CAT, GSH/GSSH, and BCA were purchased from Nanjing Jiancheng Institute of Biological Engineering. Ultrafiltration membranes with 1, 3, and 5 kDa molecular weight were obtained from Millipore Corp. All other reagents were analytical grade.

### 2.2 Selection of solid fermentation strains

Different *Lactic acid bacteria*, *Aspergillus oryzae* and the mixture of two kinds of strains were chosen to ferment the soybean meal by solid state fermentation. The dried soybean meal power was wetted with distilled water to maintain the moisture content 50% and then placed the sample into the autoclave at 121°C for 20 min. The sterile soybean meal was inoculated with 10% inoculum size and placed in the incubator with the temperature of 30°C to ferment 60 h. After fermentation, the fermented soybean meals were dried in oven with the temperature of 50–60°C. Dried fermented soybean meals were stored at −20°C for the determination of trypsin inhibitor, antigen protein, and soybean peptides (Fei et al., 2017).

#### 2.2.1 Determination of trypsin inhibitor (TI)

Trypsin inhibitor was measured according to the method of BNPNA, Jaw, K. S with modifications (Feng, Liu, Xu, Liu, & Lu, 2007; Feng, Liu, Xu, Lu, & Liu, 2007). The fermented soybean meals were mixed with Tris‐CaCl_2_ buffer, pH 8.2 and incubated in a water bath with 37°C to extract 1 h. The solution was centrifuged for 10 min with 3555 g. 5 mL 0.04% BNPNA was used as the substrate to be added to the solution to start the reaction in 37°C for 10 min and then added 1.0 mL 30% acetic acid to end the reaction, which the reaction system was 10 mL. One unit of trypsin inhibitor activity was defined as the decrease of 0.1 absorbance units at 410 nm.

#### 2.2.2 Determination of antigen protein

The antigen protein in soybean meal mainly contained soybean glycinin and β‐conglycinin, which were extracted with 0.03 mol/L Tris–HCl buffer, pH 8.0, containing beta‐mercaptoethanol 0.7 mL. The supernatant after centrifuging was collected to determent the content of soybean glycinin and β‐conglycinin with the ELISA kits.

#### 2.2.3 Determination of soybean peptides

The soybean meal was fermented with different strains included *Lactic acid bacteria*, *Aspergillus oryzae* and the mixture strains of *Lactic acid bacteria* and *Aspergillus oryzae*, and the peptides were determined according to the method of Lowry with modifications (Jaw et al., 2007). The specific operation was as follows. The fermented soybean meal powder was added to moderate distilled water to extract peptides in a water bath at 30°C for 1 h. The extracting solution was centrifuged for 15 min with 4000 rpm in a refrigerated centrifuge at 4°C. The supernatant was added to the equivalent 15% trichloroacetic acid (TCA) to precipitate the macromolecule protein and the supernatant was used to determinate the content of soybean peptides. The absorbance of supernatant was measured by the method of Folin‐phenol with ultraviolet spectrophotometer, and reductive glutathione was regarded as the standard.

### 2.3 The activities of proteinase during the fermentation

The fermentation time was confirmed firstly according to the enzyme activities and the content of peptides during the fermentation. The enzyme activities were determined according to the method of Folin‐phenol method based on GB/T 23527‐2009, which regarded casein as the substrate. The activities of acid proteinase, neutral proteinase and alkaline proteinase were determined during the fermentation with different buffer solution of pH 3.6, 7.5, and 11. 1 mL enzyme diluent was added to 1 mL 2% casein to react 10 min accurately at 40°C and then added 2 mL 0.4 mol/L TCA to terminate the reaction for 20 min. After centrifugation, 1 mL supernatant was added to 5 mL 0.4 mol/L Na_2_CO_3_ and 1 mL Folin reagent. The absorbance of the reaction system was determined at 680 nm. The definition of one unit enzyme activity is the enzyme content in 1 mL enzyme solution that producing 1 μg tyrosine by hydrolyzing casein for 1 min at 40°C.

### 2.4 Optimization of fermentation conditions for preparation the soybean peptides

According to the single factor experiments, the ratio of material to solvent, inoculum size and inoculation ratio of *Lactic acid bacteria and Aspergillus oryzae* were selected as the influence factors to optimize the fermentation conditions for preparation soybean peptides. Response surface methodology (RSM) of Box–Behnken was designed by Design‐Expert 8.0 software to optimize fermentation conditions, which regarded the yield of soybean peptides as the response value.

### 2.5 SDS‐PAGE electrophoresis

SDS‐PAGE was performed based on the method of Laemmli with modification (Issoufou et al., 2010). The sample was treated with sample buffer contained β‐mercaptoethanol (1:10) and heated in 95°C water bath for 5 min. After heating, the mixture was centrifuged at 4440 g for 10 min to obtain the supernatant. The discontinuous vertical plate gel electrophoresis was conducted with 12% separating gel and 5% stacking gel, respectively. 10 μL treated sample solution and standard proteins were added to the corresponding aperture in the preparative gel. The sample in the stacking gel with constant voltage of 80 V until the sample turned into the separating gel while the voltage was turned to 120 V. After electrophoresis, the gels were stained with Coomassie Blue G‐250 for 2 h and then decolorated with methyl acetate decoloring liquid.

### 2.6 Ultrafiltration

The soybean peptides were extracted from the fermented soybean meal. Then, the soybean peptides solution was filtered by ultrafiltration apparatus with different molecular weight membrane of <1, 1–3, 3–5 and >5, and the peptides were cut off to obtain four components (I, II, III and IV). The four components were froze drying respectively and stored at −20°C for further study.

### 2.7 Determination of antioxidant activity in vitro of the soybean peptides

#### 2.7.1 DPPH radical scavenging activity

DPPH radical scavenging activity was determined according to the method of Kimatu, B with modifications (Kimatu et al., 2017). 1 mL the four components sample solutions of the concentration 0–0.5 mg/mL were added to 4 mL DPPH (0.12 mmol/L) and mixed. The mixture was kept in dark incubated at room temperature for 30 min. The DPPH radical solution without sample was regarded as the control group (Ac). The absorbance value of the samples (As) and control group were measured at 517 nm, which VC was regarded as the positive group. All experiment was triplicate and compared with the unfermented sample. The DPPH radical scavenging activity (DRSA) was calculated as the following formula:

$$\text{DRSA}\%=\frac{\mathrm{Ac}–\mathrm{As}}{\mathrm{Ac}}\times 100\%.$$

#### 2.7.2 ABTS radical scavenging activity

ABTS radical scavenging activity was measured with slightly modification based on the method of Kaprasob et al. (2022). ABTS radical can be oxidized to cationic radical ABTS^+^ by potassium persulfate. The working solution was prepared with 7.4 mmol/L ABTS stock solution and equivoluminal 2.6 mmol/L potassium persulfate for 12 h without light. The working solution was diluted with PBS until the absorbance value of 0.7 ± 0.02 was obtained at 734 nm. 0.2 mL peptides solutions of different concentration 0–0.5 mg/mL and distilled water were added to 2 mL diluted ABTS radical working solution, which served as control group (Ac)and sample group (As), and the mixture reacted for 6 min in the dark at room temperature. The absorbance value was measured at 734 nm. All experiment was triplicate and compared with the unfermented sample. The ABTS radical scavenging activity was calculated as:

$$\text{ABTS radical scavenging activity}\%=\frac{\mathrm{Ac}–\mathrm{As}}{\mathrm{Ac}}\times 100\%$$

#### 2.7.3 Reducing power

The reducing power of soybean peptides was investigated according to the method of Ma juanjuan with slightly modification (Ma et al., 2018). 1 mL sample solutions of four ultrafiltration fractions with the concentration of 0–0.5 mg/mL were diluted with 2.5 mL 0.2 mol/L phosphate buffer and mixed with 2.5 mL 1% potassium ferricyanide. The mixture was incubated for 20 min at 50°C, and then 2.5 mL 10% TCA was added to end the reaction for 10 min. Thereafter, the 2.5 mL reaction solution was combined with 2.5 mL distilled water and 1.0 mL 0.1% FeCl_3_ to react for 10 min at room temperature. The solution was centrifuged for 10 min at 4000 rpm and the absorbance of the supernatant was measured at 700 nm. VC was regarded as the positive control group.

### 2.8 Activity of inhibition of erythrocyte oxidative hemolysis

#### 2.8.1 Assay for erythrocyte hemolysis

The erythrocyte oxidative hemolysis protection of peptides on AAPH‐induced was investigated based on the method of Wenzhen Liao (Liao et al., 2014). Erythrocytes were obtained by centrifuged at 1200 *g* at 4°C for 10 min from blood sample which was collected from healthy adult volunteers and washed three times with PBS (pH 7.4). The erythrocytes were mixed with 4‐fold PBS to obtain the erythrocyte suspension. 200 μL erythrocyte suspension was added to 200 μL PBS or the soybean peptides with different concentration. The mixture was gently incubated for 30 min at 37°C and then 400 μL 0.2 mmol/L AAPH was added to incubate for another 2 h. After incubation, the reaction solution was diluted with 3.2 mL PBS and centrifuged for 10 min at 1200 *g* at 4°C. The absorbance of the supernatant was measured at 540 nm which regard as the sample treated group. To achieve the 100% hemolysis, the ultrapure water was added to the suspension. All experiments were performed in triplicate. The hemolysis inhibition rate was calculated as the follow:

$$\text{Hemolysis inhibition rate}\%=1–\frac{\mathrm{OD}540\;\text{of sample treated group}}{\mathrm{OOD}540\;\text{of}\;100\%\text{hemolysis}}\times 100\%$$

#### 2.8.2 Determination of intracellular ROS

The intracellular ROS content was measured according the previous study with appropriate modification (Suo et al., 2022) The erythrocytes were treated as described above, and then the supernatant was removed by washed 3 times after incubation and the erythrocytes were diluted with 5–fold PBS. 100 μL cell resuspension was used to determine the yield of intracellular ROS. After centrifugating for 10 min at 1200 *g* at 4°C, 200 μL of 10 μmol/L DCFH‐DA was added to incubated 25 min at 37°C in dark. After incubation, the solution was washed with PBS, and then resuspended with 600 μL PBS. The fluorescent intensity of the erythrocytes was measured by a fluorescence microplate at excitation wavelength and emission wavelength of 485 nm and 525 nm.

#### 2.8.3 Determination of MDA, GPX, SOD, CAT, and GSH/GSSH

The cell resuspension was treated as the method of 2.8.2, the suspension was centrifuged and removed the supernatant, and then the erythrocytes were added to double distilled water at ice‐water bath for 10 min and centrifuged to obtain the cell lysis buffer (Wang, Chen, et al., 2017). The content of protein, GSH and MDA were measured by kits of Bicinchoninic Acid (BCA), GSH and Microscale Malondialdehyde (MDA), respectively. The Cellular Glutathione Peroxidase Kit, Total Superoxide Dismutase Assay Kit and Catalase Assay Kit were used to determine the activities of GSH‐Px, SOD and CAT based on the instructions.

### 2.9 Statistical analysis

All the experiments were performed in triplicate and the data were expressed with mean ± standard deviation (SD), which were analyzed by the SPSS 21 software. One analysis of variance was analyzed based on Duncan's multiple range test for the determination of significance between the parameters, while the *p* value of <.05 was considered to be significant.

## 3 RESULTS AND DISCUSSION

### 3.1 Selection of solid fermentation strains

*Lactic acid bacteria*, *Aspergillus oryzae* and the mixed of the two bacterias were used to ferment soybean meal for producing peptides. *Lactic acid bacteria* can promote nutrient digestion and adsorption. *Aspergillus oryzae* can transfer the protein to peptides and degrade some anti‐nutrients (Yuan et al., 2017). The yield of soybean peptides, removal rate of trypsin inhibitor and antigen protein were considered to evaluate the optimal strains as the indexes. The results were showed in Table 1. Compared with the single bacteria, mixed *Lactic acid bacteria* and *Aspergillus oryzae* have significant effects on trypsin inhibitor and antigen protein (*p* < .05). The removal rate of trypsin inhibitor and antigen protein reached 98.6% and 51%, respectively. This may because the synergistic effect of the two strains on the degradation of components in soybean meal (Yuan et al., 2017). It could improve the protease activities during fermentation and then increased the yield of peptides simultaneously. Therefore, the mixed *Lactic acid bacteria* and *Aspergillus oryzae* was chosen to ferment the soybean meal for improving the nutrition value.

**TABLE 1:** Effect of strains on the soybean peptides, trypsin inhibitor and antigen protein.

Strains	Unfermented	Lactic acid bacteria	Aspergillus oryzae	L:A 2:1
Peptides%	9.776 ± 0.33^a^	24.46 ± 0.88^c^	19.53 ± 0.70^b^	25.81 ± 0.66^c^
TI %	7.129 ± 0.26^a^	2.202 ± 0.15^b^	0.45 ± 0.02^c^	0.10 ± 0.01^d^
Removal rate of TI	–	69.37 ± 0.85^a^	93.67 ± 0.94^b^	98.60 ± 0.95^c^
Glycinin mg/g	37.88 ± 2.06^a^	16.83 ± 0.98^c^	19.99 ± 1.46^b^	15.85 ± 1.83^c^
Removal rate of glycinin%	–	55.57 ± 0.52^b^	47.23 ± 0.29^c^	58.21 ± 0.12^a^
β‐conglycinin mg/g	42.78 ± 2.20^a^	30.38 ± 0.04^b^	29.49 ± 1.16^b^	23.74 ± 1.31^c^
Removal rate of β‐conglycinin %	–	28.98 ± 0.99^c^	31.07 ± 0.47^b^	44.51 ± 0.40^a^

*Note:* Statistical differences between the soybean peptides, trypsin inhibitor and antigen protein in soybean meal when it was fermented with different strains are noted by different superscript letters (*p* < .05).

### 3.2 Optimization of fermentation conditions for preparation the soybean peptides

#### 3.2.1 Effect of fermentation time on fermentation of soybean meal

**FIGURE 1:**
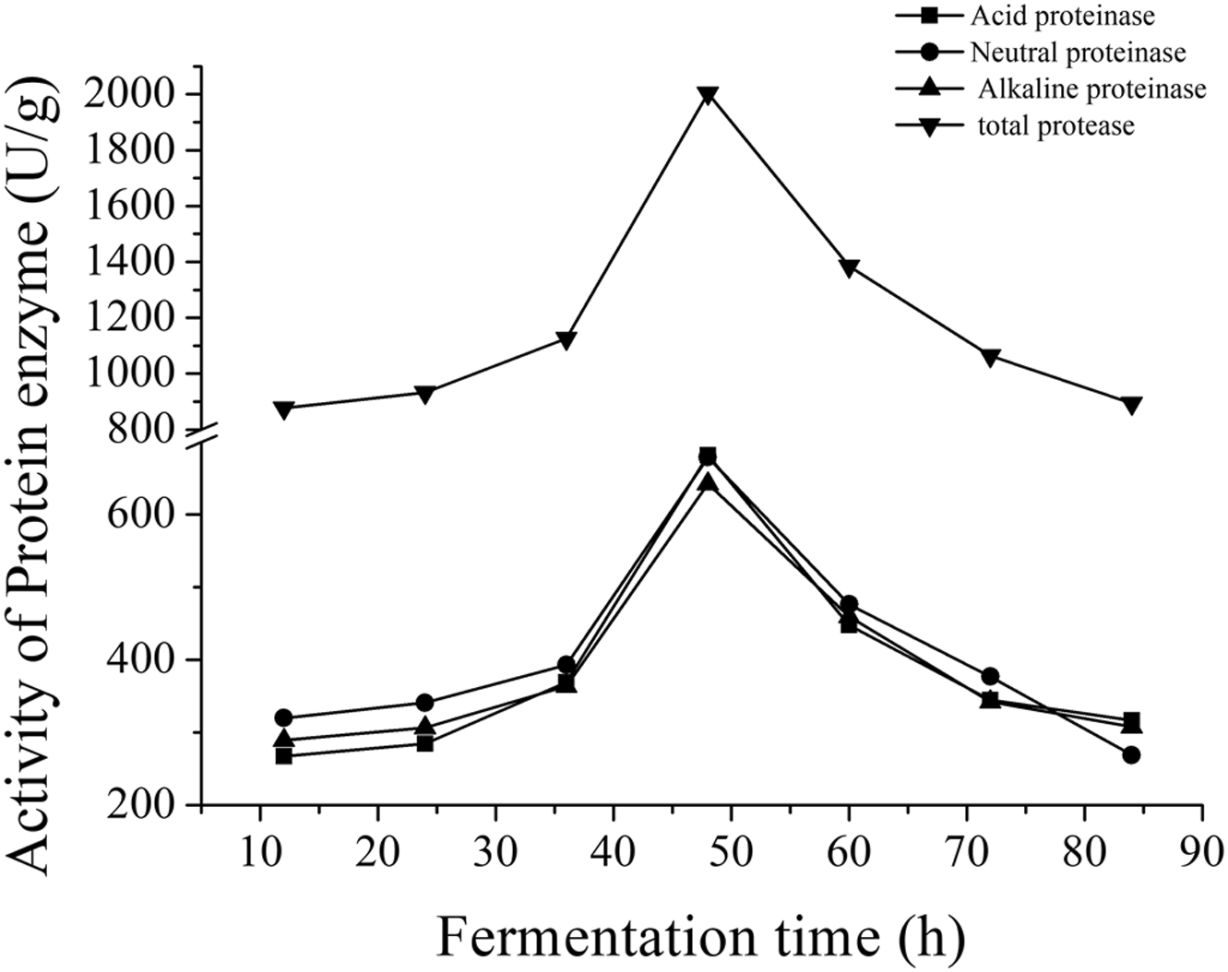
Effect of fermentation time on the proteinase activities.

**FIGURE 2:**
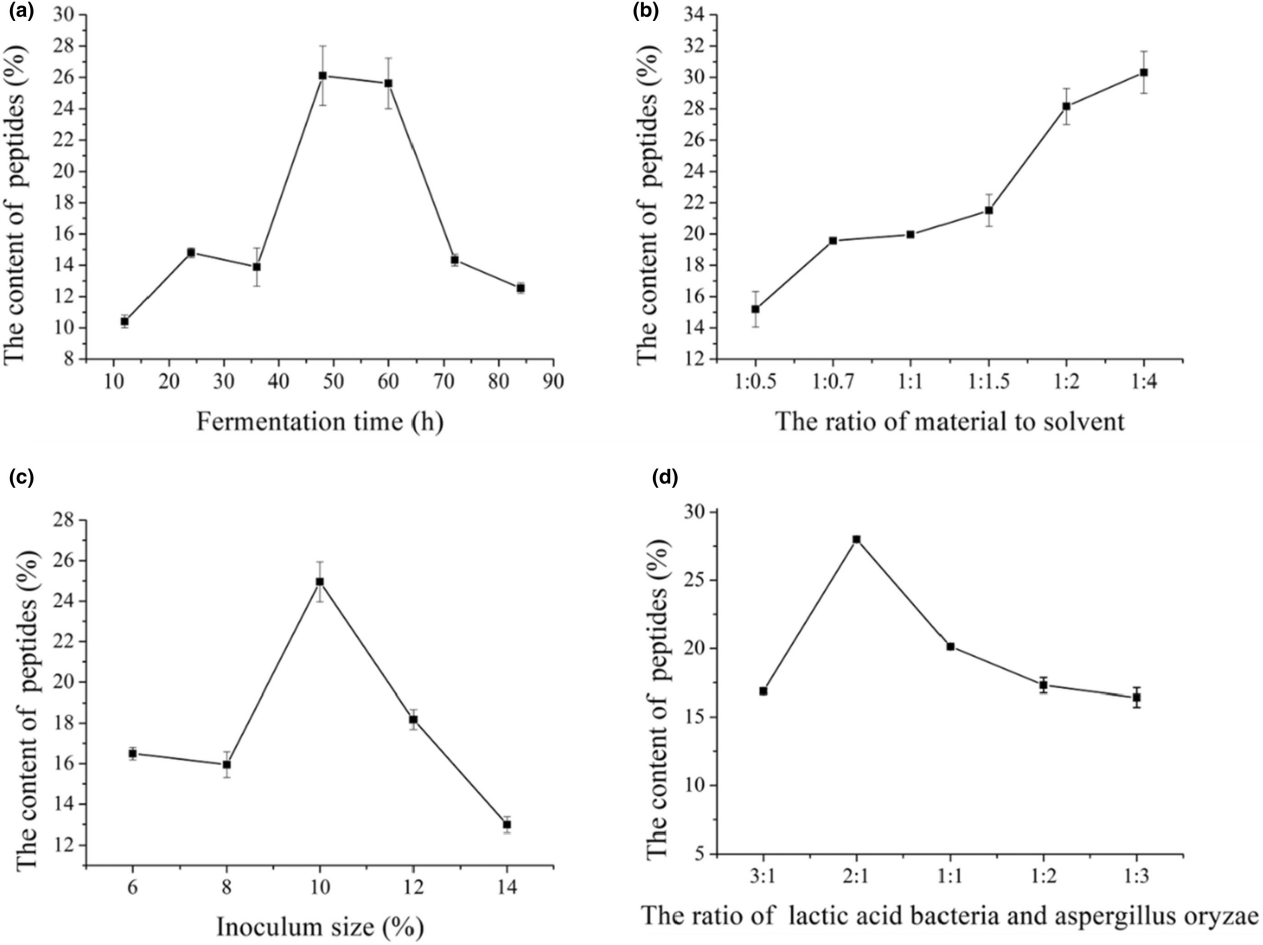
Effect of single factors on the content of soybean peptides (a: fermentation time; b: the ratio of material to solvent; c: inoculum size; d: the ratio of *Lactic acid bacteria* and *Aspergillus oryzae*).

Soybean protein could be degraded by the proteinases produced during mixed fermentation. The effect of fermentation time on the content of soybean peptides and proteinases activities were measured, as shown in Figures 1 and 2a. Figure 1 showed the activities of acid proteinase, neutral proteinase and alkaline proteinase, which had no significant difference among the three proteinases during fermentation (*p* > .05). In contrast, the proteinases activities reached highest at 48 h and had significant difference (*p* < .05) than other times, corresponded with the content of peptides (Figure 2a). After fermenting 48 h, the proteinases activities and content of peptides decreased with the increased fermented time. This may because the microorganisms begin to decline after 48 h and is not conductive to the production of proteinases. The higher proteinases activities can promote the degradation protein and increase the content of peptides. According to the results, fermentation 48 h was selected as the optimal time for the further study.

#### 3.2.2 Effect of the ratio of material to solvent on the content of soybean peptides

Effect of the ratio of material to solvent on fermentation was investigated, and the content of soybean peptides was regarded as the evaluation index. The results were presented in Figure 2b. The content of soybean peptides increased with the increasing ratio of material to solvent, while the content of soybean peptides had no significant difference at the ratio of material to solvent 1:2 and 1:4 (*p* > .05). The soybean meal cannot be wetted completely when the ratio of material to solvent was lower, and then the microorganisms cannot utilize the soybean meal for fermentation. Therefore, the ratio of material to solvent was selected 1:2.

#### 3.2.3 Effect of inoculum size on the content of soybean peptides

Inoculum size was an important factor influencing fermentation. The content of soybean peptides was presented in Figure 2c. The content of soybean peptides increased with the increasing inoculum size, and then decreased significantly (*p* < .05), which reached the highest content at 10%. Soybean meal cannot be degraded completely by the fewer strains with the lower proteinase contents, and then the content of peptides was lower. This may be attributed to the insufficient dissolved oxygen during fermenting under the inoculum size of the mixed strains exceeded 10%.Therefore, the content of soybean peptides was influenced no matter the inoculum size was higher or lower.

#### 3.2.4 Effect of ratio of *Lactic acid bacteria* and *Aspergillus oryzae* on the content of soybean peptides

Figure 2d showed the effect of ratio of *Lactic acid bacteria* and *Aspergillus oryzae* on the content of soybean peptides. The production of soybean peptides was attributed to the synergistic effect of the mixed strains. The content of soybean peptides increased first and then decreased with the increasing *Aspergillus oryzae* significantly (*p* < .05). The content of soybean peptides reached 28.01% with the ratio of *Lactic acid bacteria* and *Aspergillus oryzae* 2:1. This may because the two strains would compete growth during fermentation and inhibit each other to decrease the ability of degradation of soybean meal, which induced the content of peptides. Combined with selection strain in Table 1, *Aspergillus oryzae* could degrade the antinutritional factors in soybean meal to a certain degree. Therefore, the ratio of *Lactic acid bacteria* and *Aspergillus oryzae* with 2:1 was chosen to ferment soybean meal.

#### 3.2.5 Response surface methodology analysis

According to the single factor experiment, the ratio of material to solvent (*A*), inoculum size (*B*) and the ratio of *Lactic acid bacteria* and *Aspergillus oryzae* (*C*) were selected as the factors to optimize the fermentation conditions. The design of RSM and the results were listed in Table 2. The analysis of variance (ANOVA), the fit, and the adequacy of the models were summarized in Table 3. *p* < .05 indicated that the factors have significant effects on the content of soybean peptides produced by fermentation. *p*‐value .0033 < .05 showed that the model terms were significant, and lack of fit .1198 > .05 indicated that experimental results had a good fit with the model (*R* ^2^ = .9673). As shown in Table 3, all independent variables (*A*, *B* and *C*), quadratic of *A* ^2^ and the all interaction (AB, AC and BC) significantly influenced the content of soybean peptides. The final predictive equation of the content of peptides was obtained by the means of neglecting the insignificant factors, as shown below:

$$\begin{array}{c} Y=+29.92+2.64\times A+4.05\times B+1.5\times C+3.26\times A\\ \times B–2.88\times A\times C–2.77\times B\times C–4.17\times A2 \end{array}$$

**TABLE 2:** The RSM design of fermentation for the content of soybean peptides (Y).

Round	*A*	*B*	*C*	*Y*%
1	−1(1:1.5)	1(12)	0(2:1)	23.21
2	0(1:2)	−1(8)	1(1:1)	30.39
3	1(1:2.5)	0(10)	−1(3:1)	29.28
4	0	−1	−1	20.26
5	0	0	0	29.27
6	0	0	0	30.65
7	1	0	1	24.94
8	1	1	0	36.37
9	0	1	−1	35.14
10	0	0	0	29.59
11	−1	0	−1	19.61
12	0	1	1	34.18
13	−1	0	1	26.80
14	−1	−1	0	22.87
15	1	−1	0	22.98

**TABLE 3:** Variance analysis of regression model of Peptides.

Source	Sum of squares	df	Mean squarce	*F*‐value	*p*‐value prob > *F*
Model	379.17	9	42.13	16.45	.0033
A	55.55	1	55.55	21.69	.0055
B	131.17	1	131.17	51.23	.0008
C	18.05	1	18.05	7.05	.0452
AB	42.57	1	42.57	16.62	.0096
AC	33.24	1	33.24	12.98	.0155
BC	30.76	1	30.76	12.01	.0179
A2	63.84	1	63.84	24.93	.0041
B2	1.7	1	1.7	0.67	.4518
C2	1.01	1	1.01	0.4	.5566
Residual error	12.8	5	2.56		
Lack of fit	11.76	3	3.92	7.51	.1198

*Note*: *R* ^2^ = 96.73%, CV = 5.78%.

**FIGURE 3:**
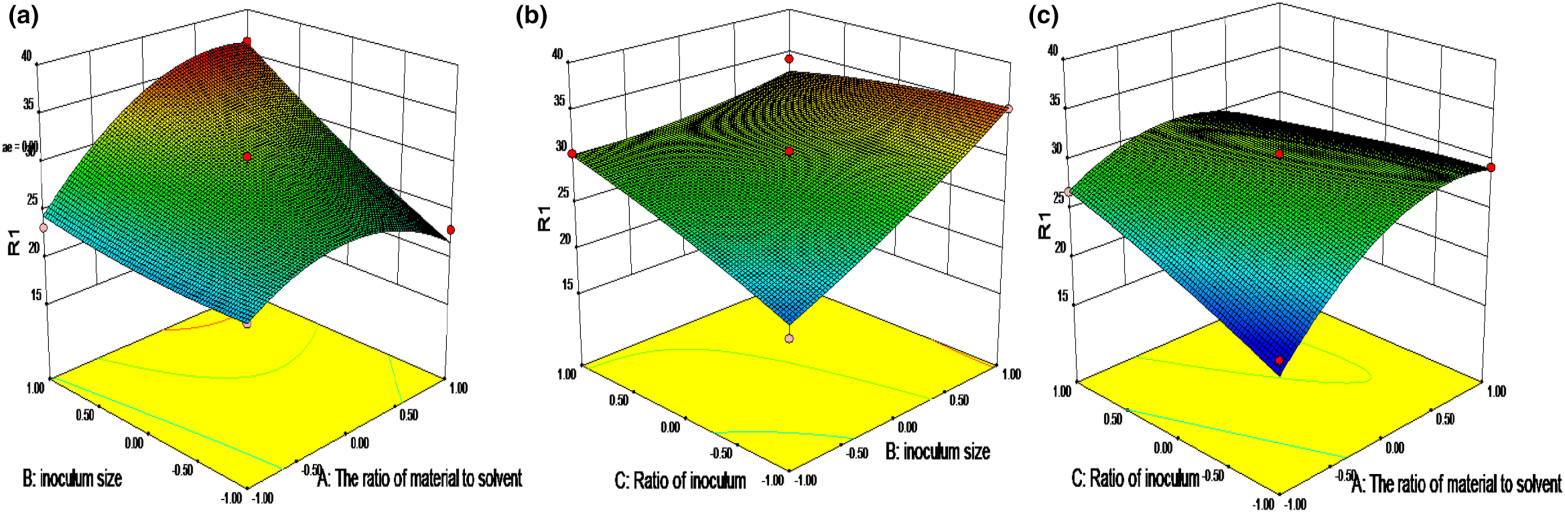
The 3D response surface plots (a: the effect of the ratio of material to solvent, b: the effect of inoculum size and ratio of *Lactic acid bacteria* and *Aspergillus oryzae*, c: the effect of the ratio of material to solvent and ratio of *Lactic acid bacteria* and *Aspergillus oryzae*).

According to the equation, the 3D response surface curves was plotted in order to predict the interaction of three independent variables on the content of soybean peptides, which were presented in Figure 3a–c. Effects of two variables were studied on the content of soybean peptides when the other variable was kept at zero level. Figure 3a indicted the effect of the ratio of material to solvent and inoculum size. The content of soybean peptides increased with the increasing inoculum size and decreased with the ratio of material to solvent over 1:2. This may because increasing inoculum size can product more proteinase to degrade the protein in soybean meal (Hong et al., 2004), while the excessive solvent can decrease the dissolved oxygen in soybean meal. It was not conductive to the growth of strains. Figure 3b indicated the content of soybean peptides increased with the increasing inoculum size and ratio of *Lactic acid bacteria* and *Aspergillus oryzae*. This may be due to the increasing proteinase during fermentation, which was in agreement with the results of Liu et al. (2017). Figure 3c described the effect of the ratio of material to solvent and ratio of *Lactic acid bacteria* and *Aspergillus oryzae* on the content of soybean peptides. The tendency was same as Figure 3b.

According to the model analysis and regression equation, the optimal fermentation conditions for production soybean peptides were obtained. The ratio of material to solvent 1:2.335, inoculum size 11.90% and the ratio of *Lactic acid bacteria* and *Aspergillus oryzae* 2:1 was used as the optimal conditions for fermentation soybean meal with the fermentation time of 48 h. In this case, the predicted content of soybean peptides was 36.50%. In order to facilitate the experiment, the conditions with the ratio of material to solvent 1:2, inoculum size 12% and the ratio of *Lactic acid bacteria* and *Aspergillus oryzae* 2:1 were selected to validate the RSM model. The content of soybean peptides was 36.10 ± 0.034%, which was in accordance with the predicted result.

#### 3.2.6 SDS‐PAGE electrophoresis

**FIGURE 4:**
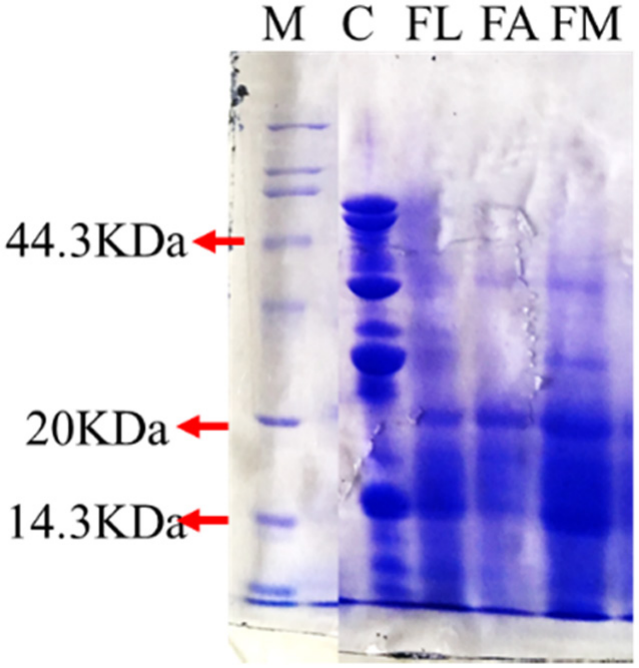
SDS‐PAGE of peptides from soybean fermented with strains. M, protein marker; C, Un‐fermentation; FL, fermentation with *Lactic acid bacteria*; FA, fermentation with *Aspergillus oryzae*; FM, fermentation with mixed strains.

The degradation of macromolecular protein in samples of unfermented, fermented with single *Lactic acid bacteria*, *Aspergillus oryzae* and mixed strains were presented in Figure 4. Compared with the Low molecular protein standard marker, the molecular weight protein over 44.3 kDa in unfermented soybean meal was degraded into <20 kDa after fermentation. The molecular weight mainly distributed under 14.3 kDa after fermented by the mixed *Lactic acid bacteria* and *Aspergillus oryzae*. According to the results, fermentation really degraded the macromolecular protein into micro‐molecule soybean peptides.

### 3.3 Antioxidant activities in vitro of ultrafiltration components

**FIGURE 5:**
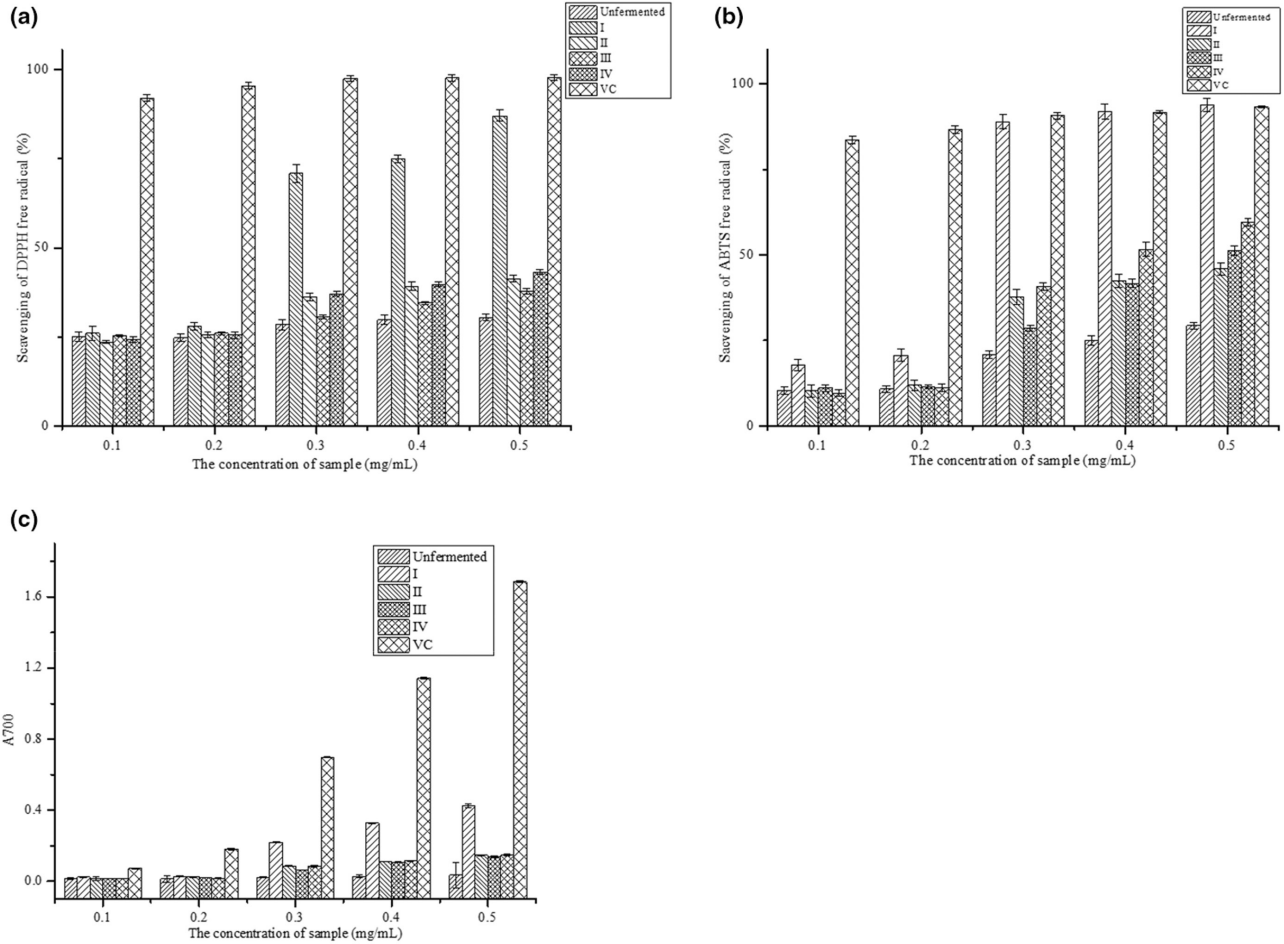
Antioxidant activity in vitro of soybean peptides. (a: DPPH scavenging activity; b: ABTS scavenging activity; c: Reduction activity).

Soybean peptides were extracted from fermentation soybean meal and were separated into four components by ultrafiltration, which was <1, 1–3, 3–5 and >5 kDa. Scavenging DPPH radical activities of the four components were evaluated at the concentration of 0.1–0.5 mg/mL. The results were shown in Figure 5a. The scavenging rate of DPPH radical increased with the increasing concentration of all peptides. Scavenging rate of DPPH radical of fermented soybean peptides was obviously higher than the unfermented peptides with a half scavenging rate (SC_50_) concentration of 0.3316 mg/mL (*p* < .05), which was lower than the positive group VC. Compared with the other components, peptides <1 kDa had the highest scavenging rate of DPPH radical with SC_50_ of 0.2517 mg/mL (*p* < .05). This may be attributed to the micro‐molecule peptides have the strong scavenging ability of DPPH radical with the exposure of antioxidant radical (Sheng et al., 2022). Besides, the peptides <1 kDa contained some substrates which could donate electrons, and convert the free radicals into stable substances and break the radical chain reaction (Wang, Lei, et al., 2017).

The scavenging ABTS radical activities of the unfermented peptides and the fermented ultrafiltration peptides with different concentration were shown in Figure 5b. The scavenging rate of ABTS radical significantly increased with the increasing concentration of samples. Compared with the unfermented, fermentation significantly improved the scavenging activity of ABTS radical under the same concentration (*p* < .05). Among the ultrafiltration components, there was a significant difference in scavenging ABTS radical. Scavenging rate of ABTS radical of component<1 kDa (I) was 93.83% at 0.5 mg/mL, which had no‐significance with VC, the positive group (*p* > .05). The half scavenging rate concentration (SC_50_) of component I was 0.2433 mg/mL, while SC_50_ of component III and IV were 0.4865 mg/mL and 0.3853 mg/mL, respectively. This may be attributed to the inhibition of ABTS^+^ generation of peptides with the lower molecular weight. The results showed that the ultrafiltration component I could eliminate ABTS radical significantly.

Reducing activity was measured based on the reduction of Fe^3+^ to Fe^2+^ with the presence of antioxidant peptides in this study, as shown in Figure 5c. Compared with the components of fermentation, the reduction activity of unfermented peptides was significantly lower than Vc and fermented peptides. There was a significant difference in reduction power between the component I and component II, III and IV (*p* < .05). This may be attributed to the exposed electrons in peptides of the different molecular weight, which can donate hydrogen to stabilize and stop radical chain reaction (Kimatu et al., 2017). The results indicated that fermentation can improve the reducing power of the peptides.

### 3.4 Activity of inhibition of erythrocyte oxidative hemolysis

#### 3.4.1 Erythrocyte hemolysis induced by AAPH

**FIGURE 6:**
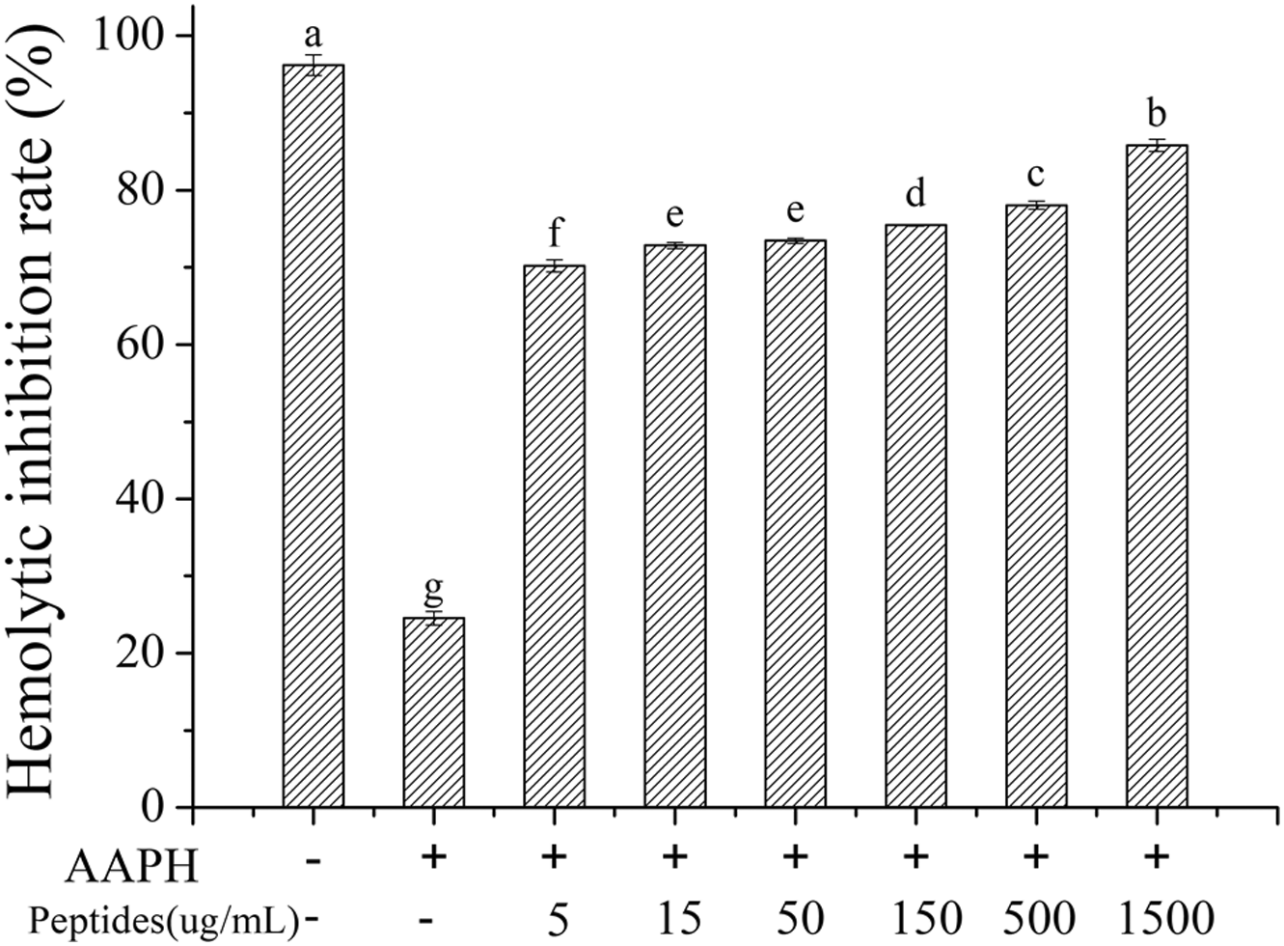
Effects of soybean peptides on AAPH induced inhibition hemolysis.

According to the experiments of antioxidant activity in vitro, the ultrafiltration component I (<1 kDa) from fermented soybean meal was selected to study the activity of inhibition of erythrocyte oxidative hemolysis. In the assay of erythrocyte oxidative hemolysis, AAPH was used as the initiator which could generate alkyl radical. The alkyl radicals can be converted to peroxyl radicals under the presence of oxygen, which can cause the destruction of erythrocyte integrity and lead to lipid peroxidation and then lead to erythrocyte hemolysis finally (Liao et al., 2014). Soybean peptides can inhibit the erythrocyte hemolysis and the inhibition rate of hemolysis was measured to evaluate the antioxidant activity of soybean peptides, which was shown in Figure 6. It indicated that soybean peptides could attenuate effectively the erythrocyte hemolysis induced by AAPH. The inhibition rate increased with the increasing peptides concentration, while the hemolysis inhibition rate reached 85.8% at the concentration 1.5 mg/mL. It indicated that soybean peptides could protect the erythrocyte oxidative damage induced by AAPH effectively.

#### 3.4.2 Effect of soybean peptides on AAPH induced ROS in erythrocyte

**FIGURE 7:**
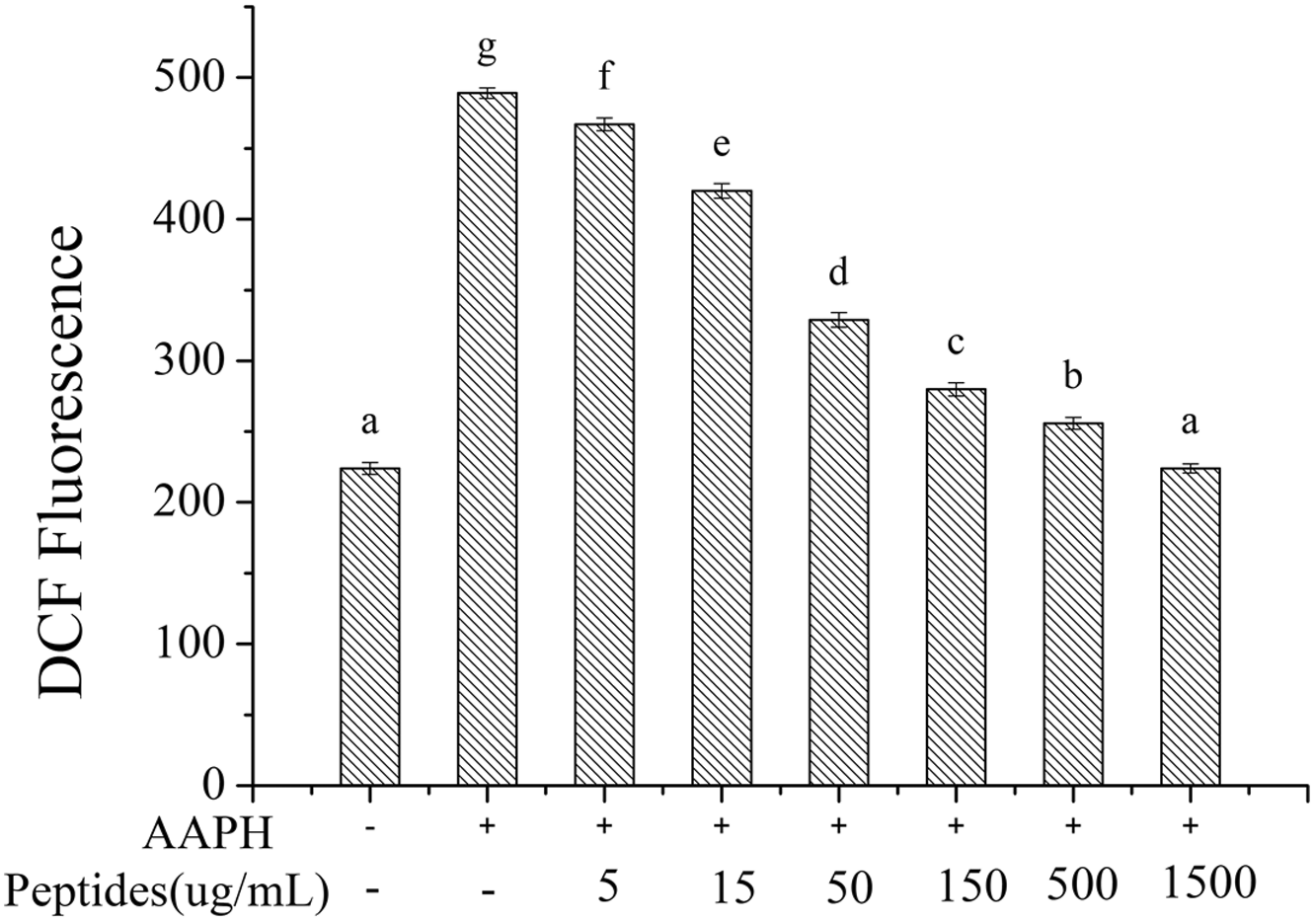
Effect of soybean peptides on AAPH‐induced ROS in erythrocytes.

ROS will generate when erythrocytes are attacked by AAPH. During ROS generation, the excessive oxidative stress will lead to erythrocyte hemolysis. In this study, intracellular ROS was measured using DCFH‐DA, a fluorescein dye that can cross cell membrane into cell plasma, where it was hydrolyzed to be DCFH by intracellular esterase. DCFH was oxidized to DCF with the presence of ROS. Therefore, AAPH‐induced ROS generation can be measured by the activity of soybean peptides to inhibit the oxidation of DCFH to form DCF. The results were shown in Figure 7. The DCF fluorescence intensity of AAPH treated erythrocytes was much higher than the normal erythrocytes, which demonstrated the AAPH treatment could produce abundant ROS. However, the DCF fluorescence intensity declined significantly with the addition of soybean peptides, which indicated that soybean peptides could inhibit the generation of ROS. The DCF fluorescence intensity in treated erythrocytes with soybean peptides at the concentration of 1.5 mg/mL had no significant difference with the normal erythrocytes (*p* > .05). Therefore, the soybean peptides can inhibit the express of ROS in erythrocytes to reduce the intracellular oxidative stress.

#### 3.4.3 Effect of soybean peptides on the activities of antioxidant enzymes, MDA accumulation and GSH/GSSH intracellular

**FIGURE 8:**
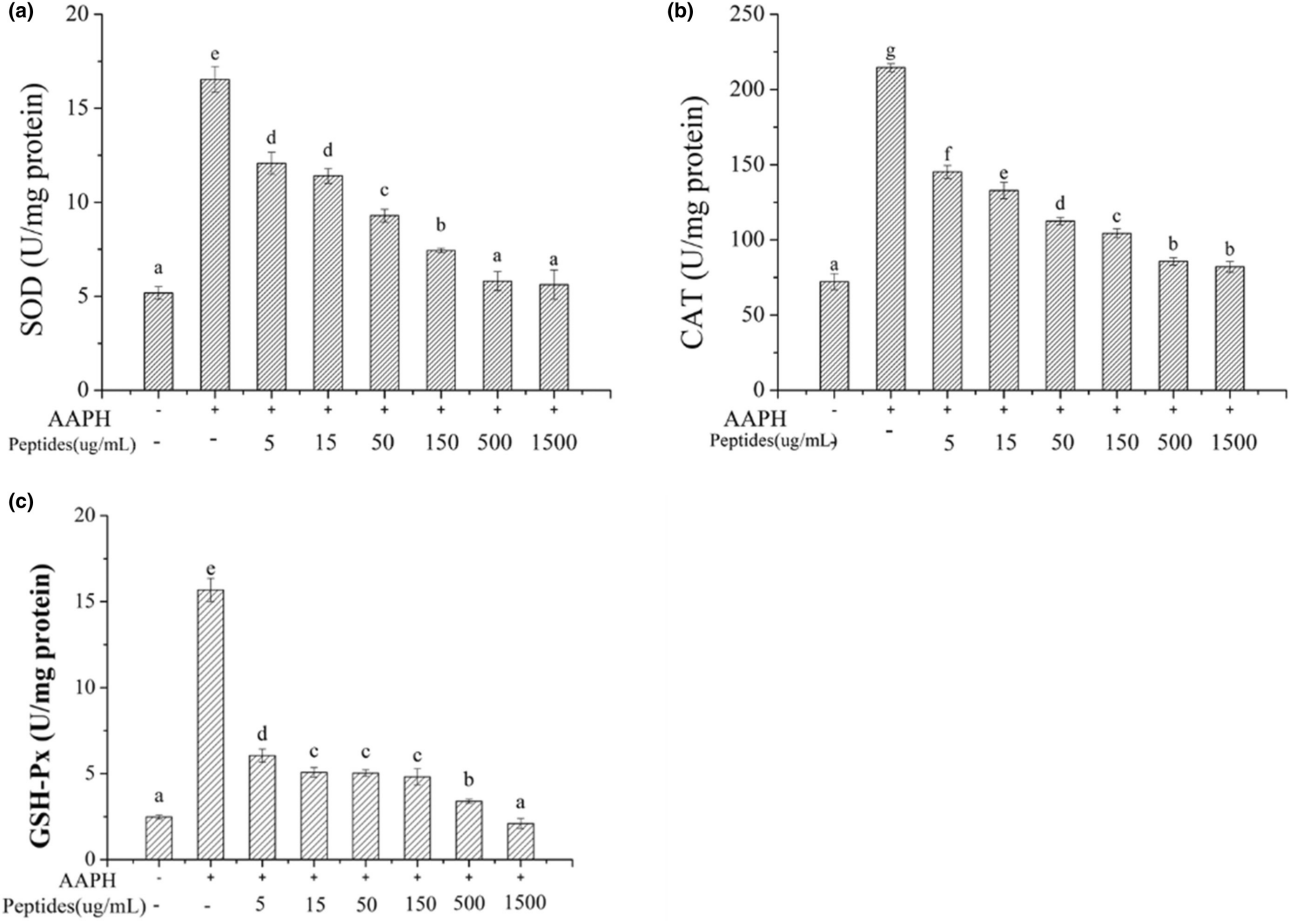
Effect of soybean peptides on activities of antioxidant enzymes in erythrocytes AAPH‐induced. a: SOD, b: CAT, c: GSH‐Px.

Oxidative stress response will occur when AAPH‐induced ROS generates, and the antioxidant enzymes intracellular include SOD, CAT and GSH‐Px will be activated to balance the oxidative stress. The activities of antioxidant enzymes will improve to scavenge the redundant radicals when AAPH attack the erythrocytes. SOD can catalyze the highly reactive superoxide anion into hydrogen peroxide (H_2_O_2_) and O_2_ with the relative low activity. CAT and GSH‐Px can decompose H_2_O_2_ into H_2_O and O_2_ and can prevent the conversion of H_2_O_2_ into more active substances. Besides, GSH‐Px will protect the cells from damage by catalyzing the reaction of hydroperoxides with GSH. The activities of antioxidant enzymes intracellular were presented in Figure 8. The activities of SOD, CAT and GSH‐Px intracellular increased significantly after the erythrocytes were treated with AAPH (*p* < .05) which indicated AAPH treatment could activated the antioxidant enzymatic defense systems intracellular. The activities of the three enzymes decreased with the increasing soybean peptides concentration based on the Figure 8a–c. When the concentration of soybean peptides was 1.5 mg/mL, the activities of enzymes were kept the level as the normal group. Therefore, soybean peptides can effectively reduce the oxidative stress in the cells induced by AAPH.

**FIGURE 9:**
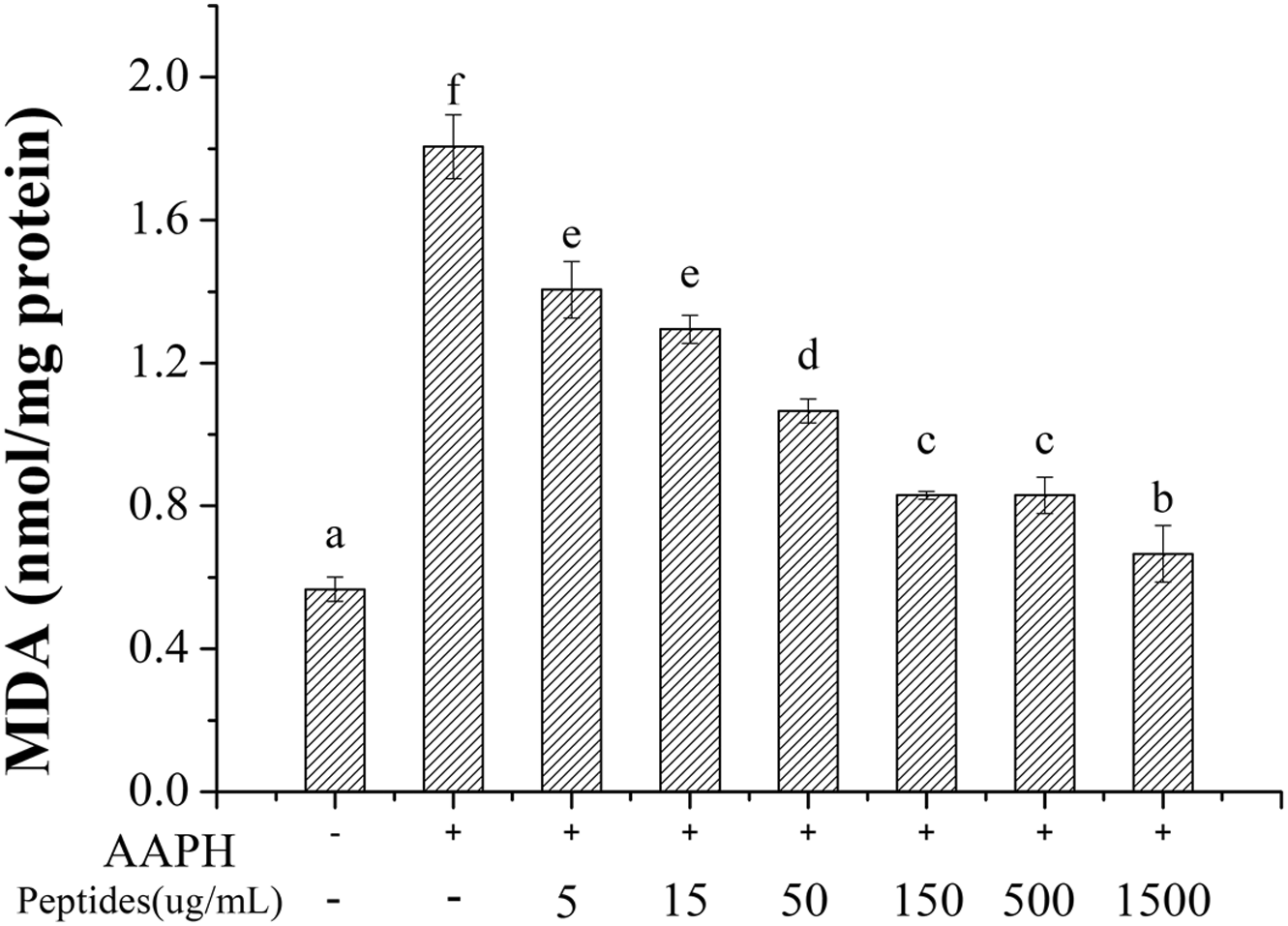
Effect of soybean peptides on the content of MDA.

Lipid peroxidation will be caused by the excessive ROS induced by AAPH and lead the release of MDA, which can destroy the cell membrane and cause cell metabolism disorder and dysfunction. Besides, excessive MDA can lead to the death of cells ultimately. As shown in Figure 9, the content of MDA in erythrocytes significantly increased from 0.67 to 2.05 nmol/mg protein with the treatment of AAPH. However, the content of MDA decreased with the increasing concentration of soybean peptides, where the content of MDA almost decreased to the control level at the concentration of 1.50 mg/mL. Thus, soybean peptides can inhibit the lipid peroxidation effectively induced by excessive ROS.

**FIGURE 10:**
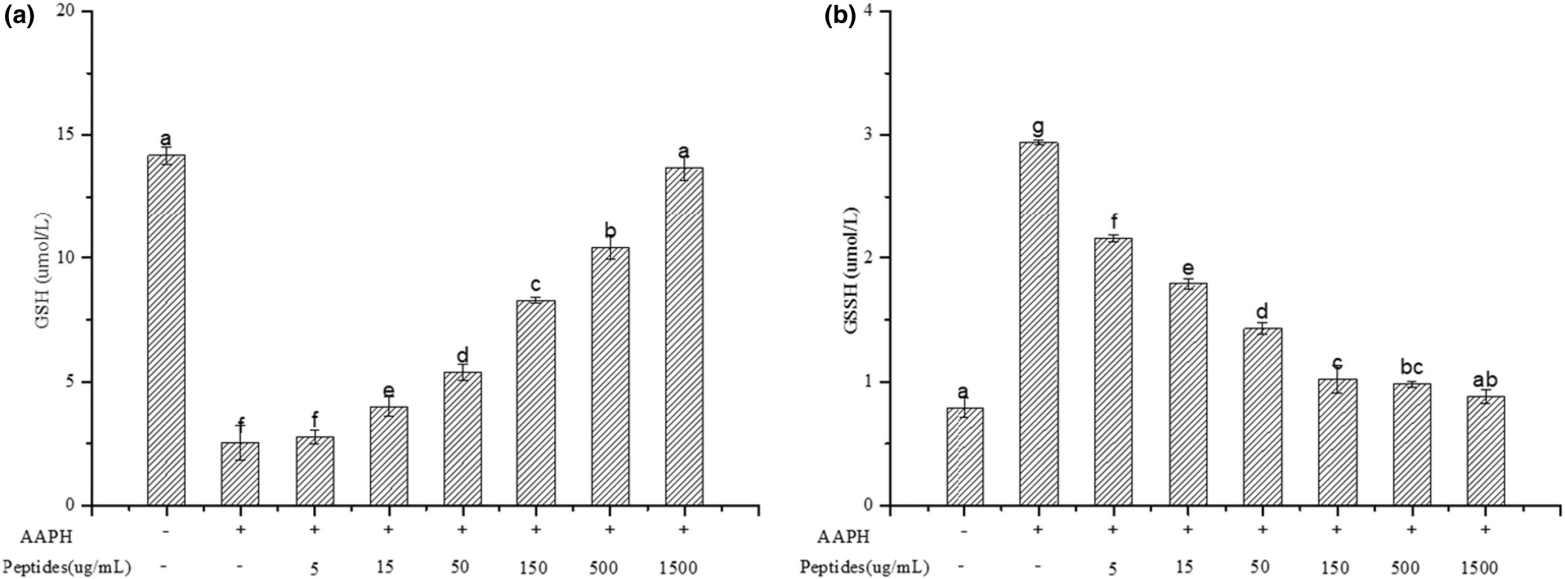
Effect of soybean peptides on the content of GSSG (a) and GSSH (b) in erythrocyte.

Erythrocytes are sensitive to the absence of GSH. GSH can scavenge radicals in erythrocytes and protect cell membrane from damage (Wang et al., 2009). Oxidative stress AAPH‐induced can decline the content of GSH, which can be oxidized into GSSH by enzymatic hydrolysis of GSH‐Px. Figure 10 showed the change of GSH and GSSH in erythrocytes. Compared with the control group, the content of GSH in treated erythrocytes with AAPH decreased significantly and the content of GSSH increased significantly (*p* < .05). However, soybean peptides significantly declined the depletion of GSH and formation of GSSH in treated erythrocytes with AAPH. The content of GSH increased with increasing concentration of soybean peptides significantly (*p* < .05) and almost reach the control level (*p* > .05). In contrast, the content of GSSH decreased with the increasing concentration of soybean peptides, which was in accordance with the change of GSH‐Px activity. This result confirmed that soybean peptides could effectively inhibit AAPH‐induced depletion of GSH in erythrocytes.

**FIGURE 11:**
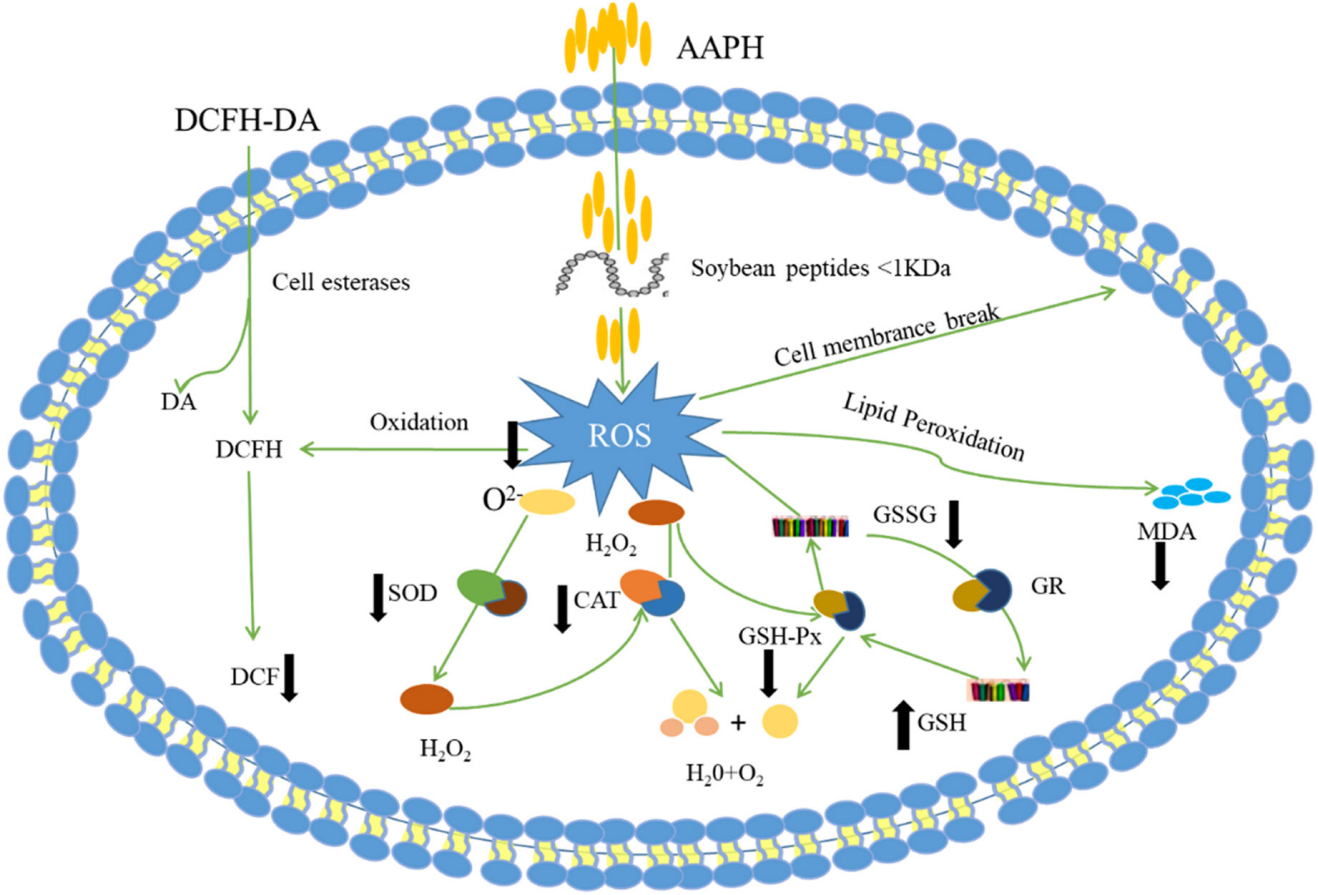
Possible intracellular antioxidant mechanisms of soybean peptides that attenuate AAPH induced oxidative stress.

In conclusion, soybean peptides can effectively attenuate oxidative stress of AAPH‐induced in erythrocytes, which mainly through inhibiting the generation of ROS and the possible intracellular antioxidant mechanisms was presented in Figure 11. Firstly, the peptides could form a protective membrane which could prevent AAPH from entering cell membranes. Secondly, the peptides directly regulate the intracellular antioxidant enzyme activities of erythrocyte induced by AAPH.

## 4 CONCLUSIONS

Soybean peptides were prepared by fermentation with mixed *Lactic acid bacteria* and *Aspergillus oryzae* from soybean meal. Based on the RSM assay, the optimal conditions for fermentation were described as the ratio of material to solvent 1:2, inoculum size 12% and the ratio of *Lactic acid bacteria and Aspergillus oryzae* 2:1, where the content of soybean peptides was 36.10%. Compared with the soybean peptides in unfermented soybean meal, the antioxidant activities of peptides in fermented soybean meal were significantly improved. After ultrafiltration, the scavenging rate of DPPH and ABTS radical of soybean peptides <1 kDa were much higher than other components, as well as the reducing power. And then the antioxidant activity intracellular of soybean peptides <1 kDa was investigated. Soybean peptides could inhibit the erythrocyte hemolysis induced by AAPH effectively, which were expressed in preventing the generation of ROS intracellular, balancing the antioxidant enzymes (SOD, CAT and GSH‐Px) system and reducing the content of MDA. In conclusion, Fermentation can degrade the macromolecule protein and some antinutritional factors in soybean meal. Besides, the soybean peptides in fermentation soybean meal can significantly improve the antioxidant activity whether in scavenging radicals or intracellular. However, the previous study was focus on the activity in vitro, which should be investigated the activity in vivo before applying the peptides in food industry. They can be developed as the additive of functional foods, medicines and cosmetic.

## AUTHOR CONTRIBUTIONS

**JuanJuan Ma:** Conceptualization (equal); formal analysis (equal); funding acquisition (lead); investigation (equal); methodology (equal); resources (lead); software (lead); validation (equal); visualization (equal); writing – original draft (lead); writing – review and editing (lead). **Keying Su:** Conceptualization (equal); formal analysis (equal); investigation (equal); methodology (equal); validation (equal). **Meimei Chen:** Formal analysis (equal); investigation (equal); project administration (equal); visualization (equal). **Shuo Wang:** Investigation (equal); project administration (equal); software (equal).

## CONFLICT OF INTEREST STATEMENT

The authors declare no competing financial interest.

## Data Availability

The data that support the findings of this study are available from the corresponding author upon reasonable request. The data are not publicly available due to privacy or ethical restrictions.
